# Synthesis and Characterization of Polyurethanes from Residual Palm Oil with High Poly-Unsaturated Fatty Acid Oils as Additive

**DOI:** 10.3390/polym13234214

**Published:** 2021-12-01

**Authors:** Javier Chavarro Gomez, Rabitah Zakaria, Min Min Aung, Mohd Noriznan Mokhtar, Robiah Yunus

**Affiliations:** 1 Department of Process and Food Engineering, Faculty of Engineering, Universiti Putra Malaysia, UPM, Serdang 43400, Selangor, Malaysia; chagoja@hotmail.com (J.C.G.); noriznan@upm.edu.my (M.N.M.); 2 Institute of Tropical Forestry and Forest Products, University Putra Malaysia, UPM, Serdang 43400, Selangor, Malaysia; minmin_aung@upm.edu.my; 3 Department of Chemical and Environmental Engineering, Faculty of Engineering, Universiti Putra Malaysia, UPM, Serdang 43400, Selangor, Malaysia; robiah@upm.edu.my

**Keywords:** bio-based polyurethanes, jatropha oil, algae oil, recovered palm oil

## Abstract

In the effort to produce renewable and biodegradable polymers, more studies are being undertaken to explore environmentally friendly sources to replace petroleum-based sources. The oil palm industry is not only the biggest vegetable-oil producer from crops but also one the biggest producers of residual oil that cannot be used for edible purposes due to its low quality. In this paper the development of biopolymers from residual palm oil, residual palm oil with 10% jatropha oil, and residual palm oil with 10% algae oil as additives were explored. Polyols from the different oils were prepared by epoxydation with peroxyacetic acid and alcoholysis under the same conditions and further reacted with poly isocyanate to form polyurethanes. Epoxidized oils, polyols and polyurethanes were analyzed by different techniques such as TGA, DSC, DMA, FTIR and H-NMR. Overall, although the IV of algae oil is slightly higher than that of jatropha oil, the usage of algae oil as additive into the residual palm oil was shown to significantly increase the hard segments and thermal stability of the bio polyurethane compared to the polymer with jatropha oil. Furthermore, when algae oil was mixed with the residual palm oil, it was possible to identify phosphate groups in the polyol which might enhance the fire-retardant properties of the final biopolymer.

## 1. Introduction

Each year around the world, a large amount of polyurethanes (PU) are produced with a wide range of applications. Polyurethanes are highly versatile materials and can be used for multiple purposes such as molding, foaming, packaging, elastomers, coating, insulating, sealing etc. causing demand for them to continuously increase. In 2020, it was reported that 24 million tons of polyurethanes (PU) were produced [1], with most of it from petroleum-based feedstock [2,3]. Unfortunately, the instability in the cost of petroleum, and the non-renewability and the non-biodegradability of these polymers, have urged researchers to find other environmentally friendly alternatives.

In the past few years, several researchers have turned back their attention to the development of biopolyurethane from vegetable oils due to straightforward processing, biodegradability and renewability of the raw material. For example, drying oils such as linseed oil [4] showed great performance for the production of biopolyurethane especially for paint coating applications due to effortless epoxidation. Semi-dry oils such as soy bean [5] and sunflower [3] was shown to produce polyurethane with similar characteristics to petroleum-based rigid PU and non-drying oils such as cottonseed oil [6], castor oil [7] and refined palm oil [8] have been used for the development of foams and elastomers by different polymerization methods with the inclusion of additives. Unfortunately, the high cost of vegetable oils (especially from dry and semi-dry oils) and the competition with edible purposes limit their full implementation.

Despite this, other sources of vegetable oils with less competition for food supply can be explored as raw materials for the development of bio-polymers. In Malaysia, the extraction of crude palm oil (CPO) from *Elaeis guineensis* produces massive amounts of by-products in the form of palm pressed fiber, empty fruit bunch (EFB), palm oil mill effluent (POME), etc. which are disposed of with a high amount of oil still within. As an example, 1.13 tons of EFB was produced per 1 ton of crude palm oil (CPO) in 2012 [9] with 3–12% of CPO remaining on the OPEFB [10]. The residual palm oil (RPO) showed similar fatty acid composition to CPO but with lower oil quality due to the formation of degradation products such as free fatty acids (FFA), peroxides, etc. [11]. Therefore, with an annual production of some 19.14 million tons of crude palm oil in 2020 [12], the EFB itself can yield about 2.5 million tons of residual oil if fully recovered. Since this residual oil is not fit for consumption due to the degradation products, its utilization to other non-food products such as bio-polyurethane has potential economic and environmental benefits.

Nevertheless, the low degree of unsaturation in RPO impede the direct usage of this material for the development of polymers. To achieve a good degree of polymerization, the vegetable oil requires a high degree of unsaturation which is measured by the iodine absorption per 100 g of oil, and this value is named iodine value (IV). The functionality and the degree of unsaturation of the resulting polyol are linked and showed to have a significant effect on the mechanical properties and stability of the resulting polymer [2]. Polyols with low functionality and high molecular weight such as from palm oil (functionality of 1.7) produce soft and more fragile polymers due to relatively lesser crosslinking in the polymeric matrix [13]. This limits it uses and hampers its full implementation. Several studies have been undertaken on converting palm oil to polyol and polyurethane using technique such as epoxidation [14,15], transesterification [16] and polycondensation [17]. However, in all these studies the final polyurethane is a mixture between petrochemical polyol and palm oil polyol which is required in order to achieve the desired strength and thermal properties. This will ultimately affect the biodegradation properties of the final polymer. Tanaka et al. [18] produced rigid polyurethanes from palm oil by glycerolysis followed by hydrolysis. They converted the triglycerides (TG) in the crude palm oil into mono-glycerides using glycerol and alcohol at high temperature. This process showed to significantly increase the functionality of the polyol by adding two hydroxyl groups instead of the untreated CPO with functionality only in the fatty acid chains in the first/second or first/third position of the TG. The final polyurethane is produced by reacting the hydroxylated oil with polyethylene glycol (PEG) or diethylene glycol (DEG) and polyisocyanate (MDI). Hence, the addition of a significant amount of petrochemical compound (PEG/DEG) is needed to produce polyurethane with acceptable mechanical properties.

Cardeño et al. [13] compared the thermal stability of alkyd resins produced from refined, bleached and deodorized soy bean oil (RBD-SBO) and mixtures of RBD-SBO-recovered frying oils, palm oil and castor oil. Their research showed that the highest thermal stability was from the mixture RBD-SBO/castor oil (70/30) followed by RBD-SBO/frying oil and RBD-SBO/palm oil. Therefore, palm oil with a low a degree of unsaturation can be blended with oils of a very high degree of unsaturation, ideally from non-food sources, to increase the functionality of the oil mix. jatropha oil and algae oil are suitable candidates for such purpose.

Jatropha oil is a non-edible, highly unsaturated oil mainly from C18:1 and C18:2 fatty acids and has been utilized for the development of biopolymers. Moreover, the oil from *Jatropha curcas* has been used for the production of polymeric adhesives not only because of the high unsaturation but also because of the high content of gums that can enhance adhesive properties [19]. Algae oil is another less explored renewable source that has shown potential to be used for the development of PU [20]. With the increasing usage of microalgae technology for the capture and reduction of CO_2_ emission, a huge amount of non-edible algae waste can be utilized for the production of biopolymers [21]. Algae oil contains a high degree of unsaturation (60–80% unsaturated fatty acids) basically from palmitoleic fatty acids (~37%, C16:1), oleic acid (~11.2%, C18:1) and linoleic acid (~40%, C18:2) [22]. Also, algae oil contains long polyunsaturated carbon chains (C22:3, C25:3) that add variation to the length of elastically active network chains and dangling chains in the polymer network to produce polyurethanes foams with similar thermal properties to foams from commercial petroleum polyols [23]. Roesle et al. [24] showed that oil from the diatom *Phaeodactylum tricornutum* has diglyceride structure with phosphate end-groups which can significantly enhance the thermal characteristics of the final biopolymer. However, the composition of algae oil vastly differs between species and growth condition. This variation can greatly affect the final mechanical and thermal properties of the polyurethane.

Therefore, our research focuses on the development of biodegradable polyurethanes from residual palm oil (RPO) with two highly unsaturated vegetable oils such as algae oil (AO) from *Chlorella vulgaris* and jatropha oil (JO) from *Jatropha curcas* as additive to produce polyurethanes. To the best of our knowledge, there is very limited study in the literature that explore the development of PU from recovered oil from palm oil waste such as empty fruit bunch and POME sludge. Residual palm oil has a different fatty acid composition, mono and diglyceride and free fatty acid contents from refined palm oil which may result in different macromolecular configuration after polymerization. Hence, this study aims to develop PU from RPO and compared that to the PU of RPO mixed with JO and AO. Since both JO and AO are highly unsaturated oils with a similar degree of unsaturation but with different composition and fatty acid structure, its effect on the thermal characteristic of the resulting polyurethane is evaluated.

## 2. Materials and Methods

### 2.1. Materials

RPO was recovered from palm oil mill effluent (POME) collected from the last sludge pit at Felda Sungai Tengi Palm Oil Mill located in Perak, Malaysia. AO was manually extracted from the dry algae powder of the species *Chlorella vulgaris* purchased from Purebulk (Los Angeles, California, CA, USA). JO was provided by Biofuel Bionas Sdn Bhd, Kuala Lumpur, Malaysia. Formic acid 98% was supplied by Friendemann Schimdt chemicals (Kuala Lumpur, Malaysia). Hydrogen peroxide 50% was purchased from R&M Chemicals (Selangor, Malaysia). Other chemical such as methanol reagent, hydrogen bromide, chlorobenzene, acetone, potassium hydrogen phthalate, phthalic anhydride, pyridine, sodium hydroxide and potassium hydroxide were reagent grade chemicals, locally purchased and used as received.

### 2.2. Oil Recovery

RPO was recovered from POME by removing water and suspended solids using a centrifuge at 7000 rpm (Centrifuge 5804, Enfield, CT, USA). The purified RPO was carefully collected from the upper layer using a pipette and was kept in the fridge at 5 °C for further usage. Dry algae powder was mixed with hexane (1:2) and homogenized using a homogenizer (Ultra turrax T25, IKA, Guangzhou, China) at 11,000 rpm for 10 min. After this, the mix was cooled down in an ice bath and was submitted to sonication for 10 min using a sonicator (Fisher Scientific, Waltham, MA, USA). The mixture of AO and hexane was separated from the solids by centrifugation at 7000 rpm for 10 min and the AO was recovered using a rotary evaporator (IKA RV10 Digital V, Guangzhou, China) at 50 °C and 150 mm of H_2_O. Finally, the process was repeated three more times to recover most of the AO.

### 2.3. Epoxidation of the Oils

Epoxidized oils were prepared from pure RPO, 90 wt% RPO and 10 wt% JO and 90 wt% RPO and 10 wt% AO by in-situ epoxidation with peroxyformic acid and sulphuric acid in a molar ratio of 0.2:0.8:2.0:0.1 (double bound: organic acid: hydrogen peroxide: catalyst) according to Dinda et al. [6]. The calculated amount of organic acid was added to 20 g of oil with specific amount of catalyst and the mixture was kept at a constant temperature of 40 °C with continuous stirring. The corresponding quantity of hydrogen peroxide was added drop wise to the mixture in order to prevent high temperature increase due to the exothermic reaction. Once the addition of hydrogen peroxide was completed, the temperature was increased to 65 °C and the reaction was carried out for 6 h. During this time, the formation of epoxy rings was monitored and recorded by measuring the OOC%. The final epoxidized oil was cooled down at room temperature and was washed using petroleum ether and distilled water. The solvent was removed from the epoxidized oil using a rotary evaporator at 50 °C and 180 mm of H_2_O.

### 2.4. Hydroxylation of the Mixture

The hydroxylation was carried out by ring opening of the epoxidized oils in the presence of methanol, water and catalyst according to Hazmi et al. [25]. Approximately 10–20 g of epoxidized vegetable oil was placed into a 100 mL three-neck flask equipped with a magnetic stirrer, a thermometer and a reflux condenser. The mixture was heated to 65 °C with constant stirring and was left to react for 2 h. After this, the mixture was transferred into a 250 mL separation funnel and was carefully washed with petroleum ether and distilled water in order to remove unreacted catalyst. Finally, the excess of water, alcohol and solvent were removed by the rotary evaporator for approximately 3–4 h. The resulting hydroxyl value and acid value of the polyols were measured and the polyols were also analyzed using FTIR and NMR.

### 2.5. Preparation of Vegetable Oil Polyurethanes

MDI (4,4′-methylene diphenyl diisocyanate) was used to prepare polyurethane for different polyols by one shot method. The molar ratio of OH group to isocyanate (NCO) was established at 1:1.6 according to the following equation:

r=WpolyolEWpolyol(WPu−Wpolyol)EWIsocyanate

where, *W_polyol_* is the weight of the polyol, *EW_polyol_* is the equivalent weight of the polyol, *W_PU_* the weight of the produced polyurethane and *EW_Isocyanate_* is the equivalent weight of the isocyanate which is 125 g/mol as supplied. The equivalent weight of the polyol was calculated as:

$$EWpolyol=\frac{56.1\times 1000}{Acid\;number\;\left(mg\frac{KOH}{g}\right)}$$

The calculated amount of polyol was manually mixed at room temperature with MDI and molded into glass Petri dishes, no catalyst or other chemical was used. Afterwards, the mixture of polyol/isocyanate was left to be moisture-cured overnight in a vacuum desiccator. Finally, the polyurethane was removed and kept in a desiccator container for further analysis. This procedure was applied for all polyols (RPO, RPO + 10%JO and RPO + 10%AO).

### 2.6. Analytical Procedures

The oxirane oxygen content was measured throughout the reaction time according to AOCS official method Cd 9-57, using potassium hydrogen phthalate instead of potassium acid phthalate. Hydroxyl value (OHv) was carried out following ASTM D 4274 99 method C-reflux phthalation and titrated with NaOH solution until reaching a persisting light pink end point. The acid measurement of the vegetable polyols was determined by the MPOB method p2.5: 2004 [26] with potassium hydroxide. Finally, phospholipids from the oils (RPO, AO, CPO) and RPO(AO) polyol were recovered according to Goh et al. [27] using methanol and purified with acid-treated fluorisin. The quantification of phospholipids was conducted using a spectrophotometer at 820 nm according to Vaskovsky et al. [28] with modified Zinzadze’s reagent.

### 2.7. Spectroscopy Analysis

The FTIR spectra was carried out using the Perkin-Elmer-spectrum with KBr plate attachment and the analysis was conducted from 4000–500 cm^−1^. ^1^H NMR was performed using an NMR spectrometer (Perkin-Elmer, LA, CA, USA) and the results were recorded at 400 MHz using JEOL Delta.

### 2.8. Gas Chromatography

Three oil samples from RPO, AO and JO were converted into the corresponding methyl esters according to the Ce 2-66 method [29], the preparation of methyl esters of fatty acids, using boron trifluoride. Then, the fatty acid composition was analyzed according to the Ce 1-62 method [30], fatty acid composition by gas chromatography, and the identification of fatty acids was undertaken using two standards from algae oil poly unsaturated fatty acid methyl ester (PUFAs) (Supelco, PA, USA) and algae oil fatty acid methyl esters (FAMEs) (Restek, PA, USA). The gas chromatography was carried out using GC-FID (Shimadzu, 2010-FID, Tokyo, Japan) and column BP-20 (SGE, Fisher Scientific, Waltham, MA, USA) with nitrogen as the carrier gas.

### 2.9. Morphological Analysis

The morphological analysis of the surface of different polyurethanes produced was conducted by scanning electron microscopy (SEM) (d4300, Hitachi, Tokyo, Japan) at 100 and 500 μm magnification.

### 2.10. Thermal Analysis of Polyurethanes

The thermal degradation of the polyurethane was verified using TGA Perkin Elmer Pyris TGA thermal analyser. Approximately, 10 mg of sample was placed into 70 μL sample holder and heated from 30 °C up to 800 °C at a heating rate of 10 °C/min under nitrogen gas flow rate (20 mL/min). DSC was undertaken using Perkin Elmer Pyris DSC thermal analyser according to ASTM E3418-03 as follows: heated from 25 °C to 120 °C at a heating rate of 10 °C/min, cooled to −40 °C at 5 °C/min, and heated again up to 120 °C at 10 C/min under nitrogen N_2_. Dynamic mechanical analysis (DMA) was carried out on DMA Q800 V20.24 with liquid nitrogen. DMA analysis was conducted according to ASTM D5062-01 standard practice. The sample of 10 × 30 × 1 mm^3^ was initially cooled to 100 °C and ramped to 140 °C at a rate of 5 °C/min and 1 Hz.

## 3. Results and Discussion

### 3.1. Characterization of the Vegetable Oils, Epoxidized Oils and Polyols

The free fatty acid (FFA) contents (%) of the RPO, JO and AO are 11.21–12.57, 2.12–2.23 and 11.85–11.91 respectively while the acid values based on palmitic acid (mg KOH/g) are 46.44–48.89, 12.4–12.75 and 46.46–48.83 respectively. The recovered palm oil has a higher FFA content compared to crude palm oil or refined palm oil which is usually below 5% and 0.1%, respectively [31]. This is obviously due to the hydrolysis and degradation of the recovered oil after exposure to the environment. On the other hand, algae oil also contain naturally high amount of FFA. The iodine value (I_2_/100 g) of the oils are 43.15–45.26, 103.62–103.69 and 116.52–118.80 for RPO, JO and AO, respectively. In comparison, the iodine values of refined palm oil is higher at around 56 due to the removal of the saturated fraction during refining [16]. The iodine values for JO were similar to results previously reported from similar oil [19]. On the other hand, The IV value for AO reported here is higher than that reported by Petrovic et al. (85 I_2_/100 g) due to the different species of algae used [32].

The fatty acids composition from the three oils used are shown in Table 1. The highest amount of unsaturated fatty acids is from AO (81.9%) followed by JO (78.91%) and RPO (47.3%). This is in agreement with the IV values stated above. However, although algae oil content of unsaturated fatty acids is only slightly higher than that of JO, AO consists of mainly polyunsaturated fatty acids (76.9%) from C18:2, C20:5 and C22:6 whereas JO contains only 33.29% polyunsaturated fatty acids mainly from C18:2 The highest content of mono unsaturated fatty acids was found in the JO with more than 45.62% from oleic acid (C18:1); for AO there is a low amount of mono unsaturated fatty acids with approximately 4.99% mostly from oleic and palmitoleic acid. Hence, the difference in the number of double bonds in a fatty acid backbone can affect the length of the dangling chain in the resulting polyurethane which consequently will affect the mechanical and thermal properties.

The fatty acid profile of algae oil also differs greatly for different species and different growing conditions [33]. The oil from *Chlorella vulgaris* contains a much longer hydrocarbon chain (C20:5) with five double bonds in its backbone. In comparison, algae oil from another *Chlorella* species reported by Pawar et al. [20] contains predominantly C18 hydrocarbon (64%) with one double bond (C18:1). Hence, there is a great variation in the chemical structure of the algae triglyceride which is known to impact the properties of the resulting polyurethane.

### 3.2. Analysis of Phospholipids

Table 2 shows the results obtained from the detection of phospholipids in RPO, AO, JO and RPO(AO) polyol. AO contains a very high concentration of phospholipids compared to JO and RPO. According to Gurr and Brawn [34], phosphate in AO from *Chlorella vulgaris* is mostly presence in the form of phosphatidyl choline from the diglycerides of C16:0, C18:1, C18:2 and C:18:3 fatty acids, which are the main reactive groups during polymerization. The addition of algae oil containing a high amount of phosphate as additive can significantly increase the thermal stability and fire-retardant properties of the final biopolymer as explained by Lewis A. [35]. However, it is apparent that the concentration of phospholipids in the polyol from RPO(AO) is significantly reduced from the original content in the AO. This reduction could be due to the formation of water-phospholipid emulsion during washing after epoxidation and hydroxylation process. The low content of phospholipids in JO and RPO indicates that only a small concentration of phospholipids will end up in the polyols and the final PU from JO and RPO.

The phospholipid content found in this study was, however, lower than that found in literatures. Nichols B. [36] showed that algae oil from *Chlorella vulgaris* contains around 30% *w/w* of phospholipid from diglycerides of phosphatidyl choline and phosphatidyl ethanolamine among others under normal light growing conditions. Similarly, Rao et al. [37] found that jatropha oil seeds contain approximately 1.4 wt% of phospholipids which is significantly higher than the value found in this study. This is possibly due to the extraction method used in this study in which hexane was used as the solvent to recover the maximum amount of oil. As hexane is a highly non-polar solvent, it is expected that less phospholipid will be extracted compared to using other more polar solvents used in the studies mentioned above [36,37].

### 3.3. H^1^ Nuclear Magnetic Resonance (NMR) Spectra of the Vegetable Oils

**Figure 1:**
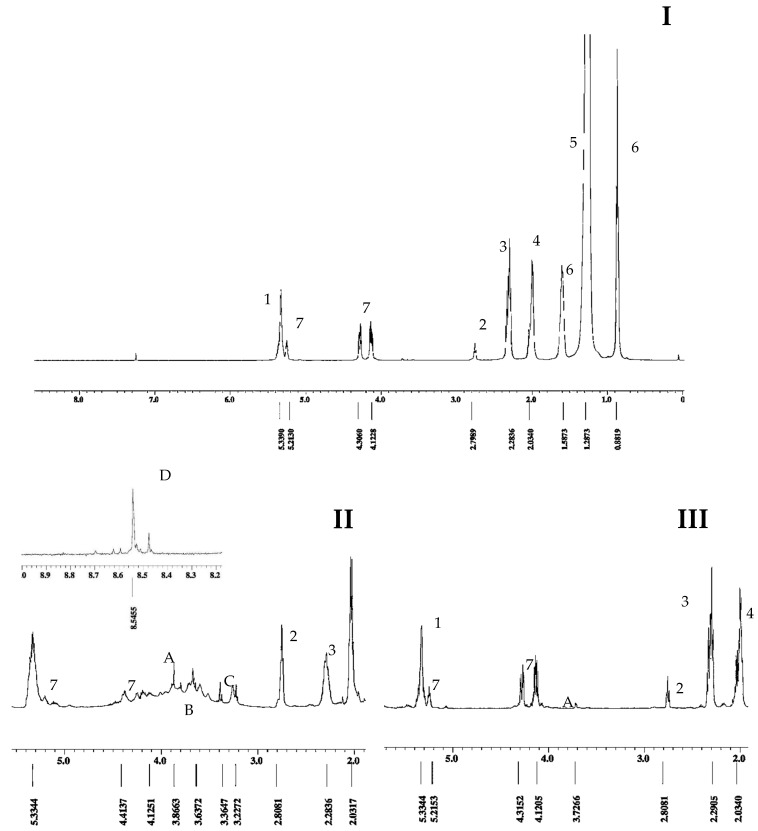
H^1^ nuclear magnetic resonance (NMR) spectrum of RPO (**I**), AO (**II**) and JO (**III**).

The proton (H^1^) spectrum of the RPO, AO and JO are shown in Figure 1 and the peaks assignation is shown in Table 3. The analysis of the signals was conducted according to Gustone F. [38], Hatzakis et al. [39] and Aung et al. [19]. Several similarities are shown in the structure of the different oils which are basically constituted of glyceride bone and acyl chains (i.e., fatty acid chains) with different unsaturation degrees. For example, in the spectrum of JO the strongest peak corresponds to alkenyl group (signal No. 1). However, in AO the peak found for double allycil groups (signal No. 2) is higher than signal No1. For AO, strong peaks were found in high amount between δ = 3.3 to 4 which are distinctive peaks from the hydrogen-choline compounds found in vegetable oil phospholipids. Unfortunately, the separation in this range was not completed because of the low solubility of phospholipid compounds with the solvent used during the analysis (i.e., chloroform). According to Hatzakis et al. [39], the peak found at 3.8 corresponds to protons on the fatty acyl chain of phosphtidylcholine (PC) and the peak found at 8.4 is related to the ^+^NH_3_ group from phosphtidylethanolamine (PDA). Hence, the H^1^ NMR for the different oils is in agreement with the analytical procedure described above in which the highest concentration of phospholipids was found in AO and zero to low amounts in RPO and JO.

### 3.4. Oxirane Oxygen Content (OOC%)

**Figure 2:**
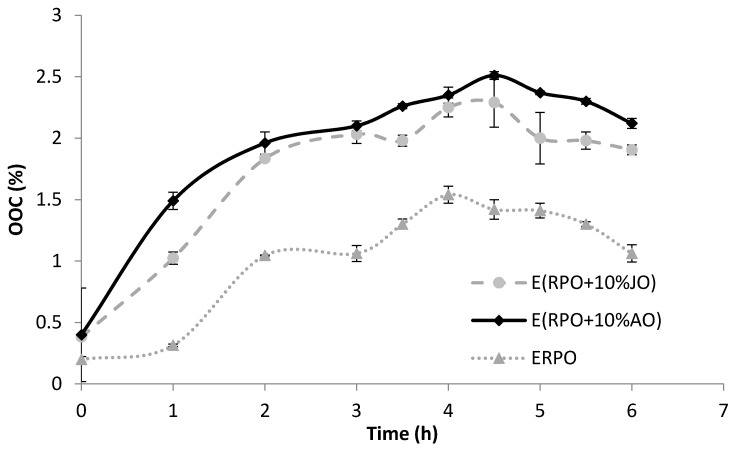
Analysis of OOC (oxirane oxygen content) during epoxidation of RPO, RPO + 10%JO and RPO + 10%AO.

The analysis of OOC% of RPO, RPO(JO) and RPO(AO) during the epoxidation process is presented in Figure 2. The graph shows the increment of epoxy groups after the inclusion of oxygen into the unsaturated bond of the different oils. The process continued until the maximum OOC was achieved and it was followed by ring opening of the cyclic ethylene oxide which led to reduction in OOC. The epoxidize RPO (ERPO) without additive showed the lowest speed of reaction, the shortest time to reach maximum OOC, and the lowest maximum OOC. This might be due to the low degree of unsaturation of the RPO. However, the addition of 10% AO and JO to the RPO significantly increase the OOC value as well as the reaction time.

Table 4 shows the characterization of residual palm-based epoxy and polyols with and without additive. Based on the result, the addition of only 10% AO and JO as additive increases approximately 40% and 30% the OOC of epoxidized RPO(AO) [ERPO(AO)] and epoxidized RPO(JO) [ERPO(JO)] compared to epoxidized pure RPO (ERPO), respectively. However, the maximum OOC values obtained in this study are lower compared to the theoretical OOC calculated using the IV value of each oil according to Hazmi et al. [25]. All the samples have similar oxirane conversion with a yield of 60 to 70% OOC. Finally, the value of IV in the epoxidized samples indicated that the epoxidation process was incomplete and that a certain number of ethilenic groups did not react and optimization of the reaction is required.

In addition, the acid value from the ERPO(AO) was shown to be higher than the ERPO(JO). Naturally, AO contains high acid value which can interfere with the polymerization due to the reaction with (N) isocyanate instead of the OH form polyol. Nevertheless, this might be minimized due to the high OH obtained from the polyunsaturated AO. Finally, the value of OH obtained from RPO was higher than values reported from untreated crude palm oil CPO (50 mg KOH/g), but, it is lower than the value reported for refined, bleached and deodorized (RBD) palm olein which was found to be 110 mg KOH/g [40]. The OH value of RPO(AO) polyol is almost double that of RPO polyol again showing that the addition of 10% algae oil increases the hydroxyl value of the polyol significantly.

### 3.5. Fourier Transform Infrared (FTIR) Analysis of Different Polyols

**Figure 3:**
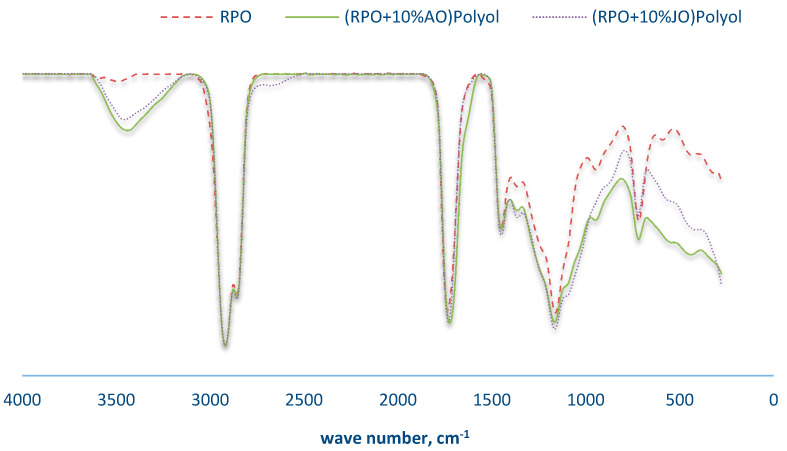
Hydroxylation of RPO, RPO + 10%JO and RPO + 10%AO polyols is described by Fourier transform infrared (FTIR) spectra.

FTIR of the epoxidized oils after hydroxylation is presented in Figure 3 and the FTIR analysis was reported according to Aung et al. [19]. The formation of polyol for all the oil samples was corroborated with the characteristic hydroxyl band observed at 3475 cm^−1^. It was found that the hydroxyl peak obtained from RPO(AO) was the highest which confirms the result of hydroxyl values in Table 3. The vibrations attributed to CH_2_ bending, -CH bends, and CH_2_, were found at 1464, 1407 and 1311 cm^−1^ absorption bands, respectively and could be attributed to the fatty acid chains of the triglycerides. It was found that the RPO(AO) polyol showed the highest area at these bands followed by RPO(JO) and RPO. This can be due to the longer fatty acid chains’ present in AO (i.e., fatty acids from C22 and C25). In addition, characteristic bands of the hydroxyl and carbonyl groups were found in the 3373 and 1750 cm^−1^ region. Finally, no apparent peak from ethylenic bonds was found at 1630–1640 cm^−1^ which might indicate that remaining double bonds could be opened during polyol reaction due to high temperature used. (e.g., 90 °C) (Lai et al., 2013).

### 3.6. H^1^ NMR Analysis of RPO(AO) Polyol Formation

**Figure 4:**
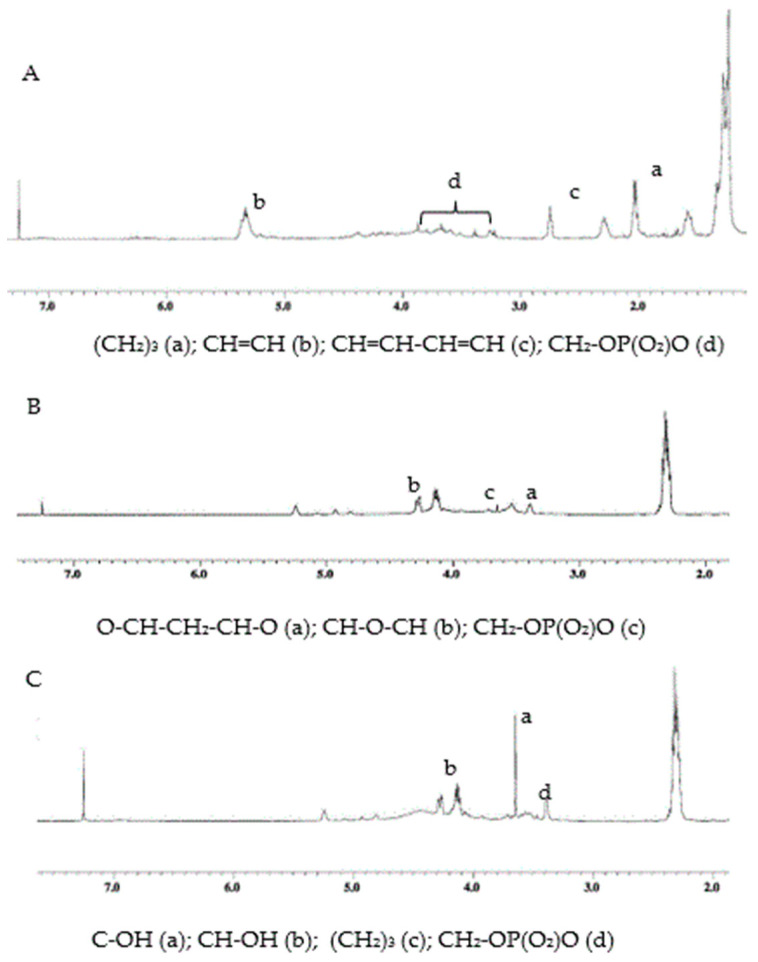
Pathway of the hydroxylation of RPO(AO) analyzed by H^1^ NMR showing the oil RPO(AO) (**A**), the epoxidized RPO(AO) (**B**) and RPO(AO) polyol (**C**).

The hydroxylation of RPO(AO) oils was corroborated by H^1^ NMR. Figure 4, shows the pathway of RPO(AO) to epoxidized oil and to hydroxylated polyol, analyzed by NMR spectra. The identification of phosphate, unsaturated, epoxy and hydroxyl groups are highlighted with less attention to other groups shown previously. As mentioned before, the structure of the different oils before processing is similar however, AO can provide phosphate groups to the final polyol and to the subsequent polyurethane. During the epoxidation and hydroxylation of the oils it is possible to identify the formation of epoxy rings at 4.3 ppm (Figure 4B) and hydroxyl carbon at 3.65 ppm (Figure 4C) which is in accordance with previous reported analysis [19]. However, in all the NMR shown it is possible to identify one small peak at δ = 5.33 of alkenyl unsaturation which indicates that the epoxidation was not complete as shown in the analysis of IV (This peak was not clear in the FTIR analysis of the RPO(AO) polyol). Finally, the protons of phosphate groups were found in the final polyol from RPO(AO) which agrees with the analysis above.

**Scheme 1:**
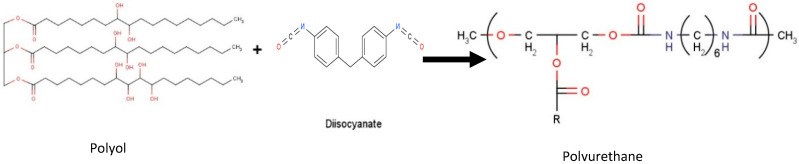
Urethane linkage.

A polyurethane linkage is created by reacting the poly isocyanate group, -N=C=O, with the hydroxyl (-OH) group in the polyalcohol (polyol) in the presence of a catalyst (Scheme 1) [41]. Hence, when more hydroxyl groups are presented in the polyol more linkages will be obtained in the polymer. Furthermore, the hydroxyl number of the polyols has a linear effects on crosslinking density of the poly-urethanes, expressed as the number of elastically active network chains per unit volume, and inversely proportional to the average molecular weight between crosslinks which resulted in better mechanical properties [4].

#### 3.6.1. Spectroscopy Analysis of Polyurethanes (FTIR)

**Figure 5:**
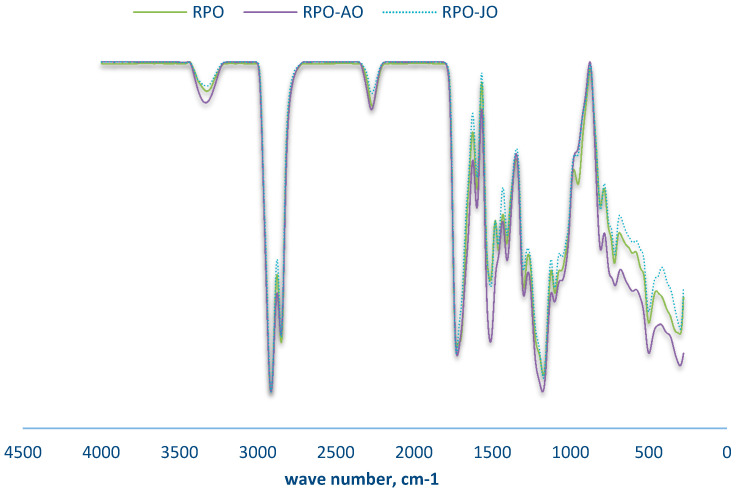
FTIR spectra of the polyurethanes produced by RPO, RPO + 10%JO and RPO + 10%AO polyol with isocyanate.

The produced polyurethanes (PU) were analyzed by FTIR spectroscopy to identify their structure and the respective polymerization. Three different PU from RPO, RPO + 10%AO and RPO + 10%JO were prepared, analyzed by FTIR according to Velencoso et al. [42] and the results are shown in Figure 5. The stretching vibrations at 3325 cm^−1^ were identified as characteristic bands of N–H from the urethanes linkages. The PU from RPO with AO as additive showed the highest intensity compared to other PU. This indicates that a higher urethane linkage was obtained with AO at the same NCO:OH ratio. The unreacted NCO compounds were identified at 2290 cm^−1^ and shown to be similar in the three samples. Stretches at 2920 and 2853 were found for the C–H methane group as asymmetric and symmetric. The large difference between the peaks in the 3325 and 2920 region indicates that the three consist mostly of semi rigid and soft PU rather than hard sections. In addition, according to Zhang et al. [43] the stretching vibration at 1029 cm^−1^ indicates the presence of P–O–C symmetric bending vibration. This peak is clearly present in PU from RPO(AO) which can be attributed to the presence of phospholipid in the AO. No peak in the bands for the OH group (3400 cm^−1^) was found which indicates that all the available OH groups reacted with the MDI to form the PU. Also, no stretch corresponding to urea bands (1628–1690 cm^−1^) was found.

#### 3.6.2. Morphological Analysis

**Figure 6:**
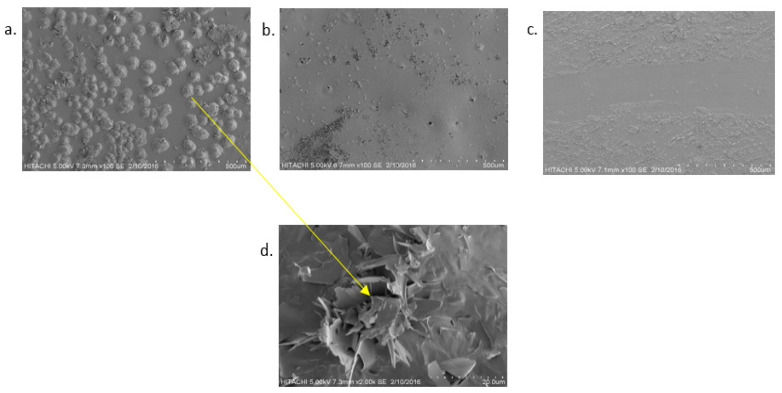
Surface of the polyurethanes using scanning electron microscopy (SEM, 5.0 KV:500 μm magnification) obtained from the polyols of (**a**) RPO (**b**) RPO + 10JO (**c**) RPO + 10%AO and (**d**) RPO with SEM (5.0 KV:100 μm magnification).

The surface of the polyurethane obtained from hydroxylated RPO, RPO + 10%JO and RPO + 10%AO was scanned by using SEM (d4300, Hitachi, Japan) and the results are shown in Figure 6. The surface of the polyurethane obtained from RPO (Figure 6a) showed an amorphous growing of several spike shapes without specific orientation (Figure 6d). The formation of amorphous spike shapes caused cracks which weaken the polymer [44]. On the other hand, the addition of polyunsaturated oil such as JO (Figure 6b) to the original RPO significantly reduced the formation of spike shapes with better orientation. A smoother surface was obtained when the RPO was blended with 10% AO (Figure 6c).

### 3.7. Polymer Thermal Analysis

#### 3.7.1. Differential Scanning Calorimeter (DSC)

**Figure 7:**
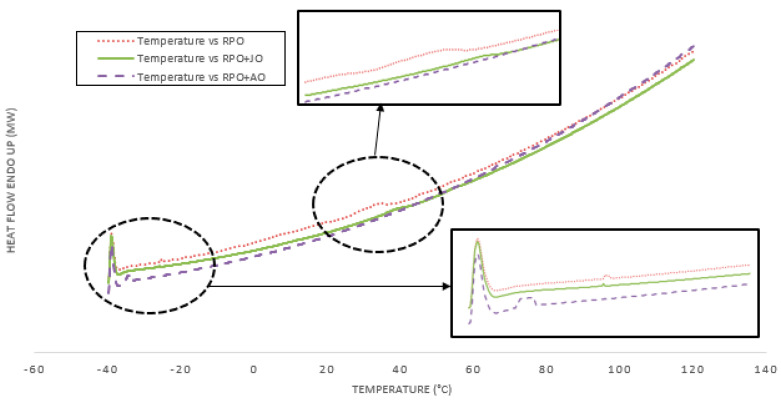
Differential scanning calorimetry (DSC) curve of polyurethanes from vegetable oils.

Figure 7 shows the analysis of DSC of the different polyurethanes synthesized while Table 5 summarizes the values obtained for T_g1_ and T_g2_ for the soft segment and hard segment of the different PU produced. The change of slope on the DSC shows that there is one glass transition temperature T_g1_ for soft segments and one T_g2_ for hard segments without melting peak, indicating that the samples have an amorphous structure [45]. The lowest T_g1_ was obtained from RPO(AO) at −33.3 °C, followed by RPO(JO) at −25.8 °C and RPO at around −25.6 °C. All samples exhibit similar T_g1_ because of the similar main source used (i.e., palm oil) and are similar to other palm-based bio polymers developed by other researchers (e.g., −33 to −15 °C) [46]. However, RPO(AO) exhibit slightly lower Tg1 possibly due to the longer fatty acid chains that have higher mobility at lower temperature.

The negative values of T_g1_ suggest that all the PUs are dominant in flexible segments provided by the long fatty acid chains of the vegetable oils. When AO is added to the RPO as additive, this might add flexibility, increase elongation at break and resistance to scratching to the final PU. In addition, the lower value of T_g1_ from RPO(AO) can also be related to the inclusion of phosphate groups in final PU. Wu et al. [47] compared the T_g_ obtained from different PU before and after the addition of tris(chloroisopropyl) phosphate (TCPP) as a flame retardant plasticizer. They obtained significant reduction of T_g_ per percentage of TCPP added as plasticizer due to the swelling of the PU structure, effect of the phosphate group and dilution of aromaticity (i.e., reaction with NCO).

The effect of hard segments in the sample and the relation with T_g2_ can be correlated with the increasing of the OH number in the polyol. All the samples exhibited hard segments due to the crosslinking with urethane groups. However, the high OH value found in the AO leads to the production of more hard segments between the polyol and the isocyanate which restricted the chain mobility and increased T_g2_.

#### 3.7.2. Thermo Gravimetrical Analysis (TGA)

**Figure 8:**
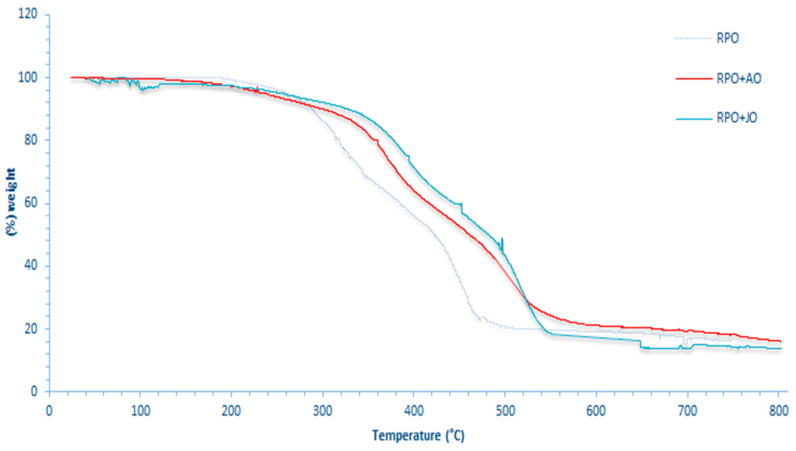
Overlay of thermogravimetric analysis (TGA) curves of the palm-based bio-polyurethanes produced.

The degradation of the PUs with temperature was analyzed using TGA and the results are shown in Figure 8. The first degradation (TD1) is associated with the loss of moisture content and suggests that the degradation of PU from RPO(AO) is slightly faster compared to other PUs due to possible water emulsion trapped in the phospholipid groups. The second onset of degradation of the PU (TD2) from all samples started at 250 to 300 °C which is associated with the hard segments on the PU [20]. It can be seen that the PU from RPO degrades much faster possibly due to higher saturation and less urethane linkage that is formed [13] and possible free isocyanate [49]. PU from RPO/JO and RPO/AO degrades at a similar rate during this stage. The third degradation stage (TD3) started at 350 °C for RPO and at 420 °C for RPO(AO) and RPO(JO). At this stage, the decomposition of the polyol soft segment occurred. The degradation rate and the percentage of weight loss of RPO(AO) is the slowest which could be due to longer fatty acid chains of AO that are squeezed out of the PU matrix during curing and the phosphate plasticizer in the AO. Hence, the usage of AO as additive was shown to significantly improve the stability of the polymer at high temperature.

Table 6 shows the degradation at 5%, 10% weight loss ratio and max residual amount of material remaining after the thermal decomposition versus the temperature measured. The initial stage of degradation (i.e., 5% weight loss) occurred at a higher temperature for the sample with RPO compared with the other polymers possibly due to less phospholipid concentration. As mentioned before, the phospholipids might have created water-oil emulsions during the epoxidation process trapped inside the polymer and not removed during the drying process of the samples. The next 10 percentage weight loss show the highest temperature for the sample with JO followed by the sample with AO, which correlates with a higher degree of polymerization when compared to RPO. Finally, the three samples flatten the curve of weight loss (Final weight) at different temperatures and with different sample char residual. The sample with AO presented the highest char residual with 20.96% possibly due to the highest concentration of phosphatide presented in the AO.

#### 3.7.3. Dynamic Mechanical Analyzer (DMA)

**Figure 9:**
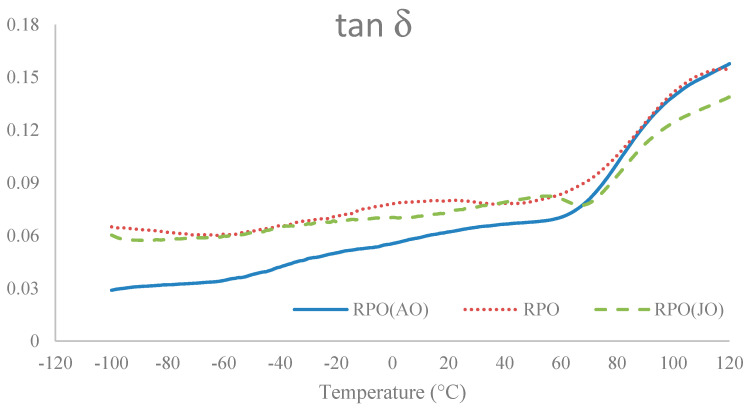
Variation of Tan d versus temperature for the different polymers.

DMA was conducted to analyze the mechanical and viscoelastic behavior of the three different materials under thermodynamic changes. Figure 9, depicts the dependence of the loss factor and the storage modulus (i.e., tan delta) with temperature. The continuous increment of tan delta might be due to accretion of the molecular chain mobility that soften the polymer at an elevated temperature.

The delta modulus (tan δ) has an inverse proportion with the OH number across the temperature range. Initially, the polymer developed with RPO(AO) showed the lowest tan δ value and that developed from RPO showed the highest. The lesser height of the delta peaks is associated with the crosslinking arising from the strong covalent bonds between the high functionality polyol and the hard segment component, which restrict the mobility of the dangling chains [50]. After the temperature of 67 °C the tan δ of the polymer from RPO(AO) increased significantly while the polymer from RPO(JO) displayed the lowest values. The restriction in the molecular mobility of the RPO(JO) due to stronger urethane linkages in the network under stress might increase the wear resistance of the PU. Hence, RPO(JO) can provide a better PU for coating application when compared to RPO and RPO(AO) [48].

## 4. Conclusions

Polyester polyols and polyurethane were prepared from recovered palm oil, recovered palm oil with 10% jatropha oil, and recovered palm oil with 10% algae oil as additive. The polymerizations were conducted via one pot epoxidation and hydrolysis followed by isocyanation of the hydroxyl groups (-OH). The characterization of the oils, epoxidized oil and polyols was conducted and the results were compared among the different samples. Residual palm oil has a much lower IV number than that of refined palm oil but a higher FFA content. Algae oil, on the other hand, has a slightly higher IV value than jatropha, but it contains predominantly C20:5 fatty acid chain which is longer and with much higher double bonds than jatropha which is predominantly C18:1. Algae oil also contains much higher phospholipid content and the phosphate groups are found to be in the polyol and the final polyurethane. The addition of 10% algae oil to RPO almost doubled the hydroxyl of value of the polyol compared to pure RPO polyol The thermal properties of the final polyurethanes were analyzed by different techniques and the results were compared. We found that the addition of polyunsaturated oil as additive into the highly saturated recovered palm oil significantly enhanced the thermal properties of the final polymer. Moreover, the polymer developed with algae oil as additive showed a higher thermal stability than that of jatropha oil due to the higher crosslinking from the polyunsaturated fatty acid chain and the inclusion of phosphate groups. Differential scanning calorimetry showed significant increment of T_g_ for the hard segment and a lower Tg for the soft segments of the polyurethanes produced with RPO(AO). Finally, the usage of, non-edible, biodegradable and renewable recovered palm oil with jatropha oil and algae oil as additive has a high potential for the production of environmentally friendly bio-based polyurethanes.

## Figures and Tables

**Table 1:** Fatty acid profile of residual palm oil (RPO), jatropha oil (JO) and algae oil (AO) obtained from gas chromatography.

Compound		Compound Name	Fatty Acid (%)		
			RPO	JO	AO
Saturated	C14:0	Methyl myristate	2.18	0.10	1.37
	C16:0	Methyl Palmitate	46.62	13.54	16.73
	C18:0	Methyl sterate	3.90	7.45	-
Mono unsaturated	C14:1	Methyl myristoleate	-	-	0.88
	C16:1	Methyl Palmitoleate	-	0.69	1.95
Polyunsaturated	C18:1n9	Methyl oleate	37.93	44.93	-
	C22:1n9	Methyl erucate	-	-	2.16
	C18:2n6	Methyl linoleate	9.37	33.29	21.05
	C20:5n3	Methyl eicosapentaenoate	-	-	52.57
	C22:6n3	Methyl docosahexaenoate	-	-	3.28

**Table 2:** Phospholipid content in different oil samples and RPO(AO) polyol.

Sample	Sample (g)	λ 820 nm	ppm
AO	1.009	1.709 ± 0.16	6237 ± 582
JO	0.909	0.518 ± 0.13	92.51 ± 17
RPO	1.015	0.812 ± 0.01	98.18 ± 0.19
RPO(AO) polyol	1.005	1.832 ± 0.35	235.7 ± 42.37

**Table 3:** Signal number, hydrogen assignation and group of oils by H^1^ NMR.

Signal	Proton	δ	Assignation of Hydrogen Atoms
1	(-CH=CH-)	5.33	All alkenyl
2	(=CHCH2CH=)	2.81	doubly allylic
3	–CH_2_CH_2_COOCH_3_	2.28	Acyl chains
4	CH_2_CH=CHCH_2_-	2.03	Allylic chains
5	(CH_2_)_n_	1.28	Acyl chains
6	CH_2_ CH_2_ CH_2_ –CH_3_	1.58/0.8	Acyl chains excluding linolenyl
7	CH_2_ –OCOR	4.12/4.31/4.41/5.20	Glycerol
A	CH_2_ –OP(O)_2_O	3.94	Glycerol/Choline
B	CH_2_ –N(CH_3_)_3_	3.81	Choline
C	-N(CH_3_)_3_	3.36	Choline
D	CH_2_ –^+^NH_3_	8.4	Anolamine

**Table 4:** Properties of the epoxidized residual palm oil (RPO), RPO with 10% algae oil (AO) and RPO with 10% jatropha oil (JO). ^1^ Value calculated according to Hazmi et al. [25].

Property	Epoxidized		
	RPO	RPO(JO)	RPO(AO)
	-	-	-
OOC_max, (%)	1.65 ± 0.11	2.33 ± 0.04	2.745 ± 0.23
Acid Value (mg KOH/g)	29.395 ± 0.47	6.46 ± 0.7	12.2 ± 1.14
IV,% per mole	17.6 ± 0.25	11.05 ± 0.28	12.43 ± 0.11
OOC Max. Theoretical ^1^	2.71 ± 0.06	3.53 ± 0.02	3.975 ± 0.35
	hydroxylated		
Hydroxyl value (mg KOH per gram)	78.525 ± 1.29	131.34 ± 2.68	152.06 ± 0.56
Equivalent weight	60.75 ± 0.5	60.92 ± 0.4	62.40 ± 0.02

**Table 5:** Glass transition temperature (T_g1_ and T_g2_) obtained under and over 0 °C, respectively, from residual palm oil (RPO); RPO with 10% algae oil RPO(AO); RPO with 10% jatropha oil RPO(JO); compared with jatropha oil (JO) reported by Saalah, S. (2016) [48] ^a^.

Sample	T_g1_	T_g2_
RPO	−25.6	27.8
RPO(JO)	−25.8	35.3
RPO(AO)	−33.3	37.5
JO ^a^	−11.45	-

**Table 6:** The 5, 10 and max percentage of weight loss versus temperatures for the samples with RPO, RPO(JO) and RPO(AO).

Weight Loss (%)	Temperature (°C)		
	RPO	RPO(JO)	RPO(AO)
5	261.33	239.54	234.10
10	288.02	312.05	305.56
max	516.23	550.34	669.53
Sample residual (%)	16.11	18.53	20.96

## Data Availability

The data presented in this study are available on request from the corresponding author.
